# Post Chikungunya Fever and Post COVID-19 Bilateral Pedal Edema: A Case Report

**DOI:** 10.7759/cureus.27588

**Published:** 2022-08-01

**Authors:** Manas Pustake, Mohammad Arfat Ganiyani, Dhwani Shah, Vijay Dhondge, Krishna Deshmukh

**Affiliations:** 1 Internal Medicine, Grant Government Medical College and Sir JJ Group of Hospitals, Mumbai, IND; 2 Global Clinical Scholars Research Training, Harvard Medical School, Boston, USA; 3 Internal Medicine, Sapphire Multispeciality Hospital, Nashik, IND; 4 Internal Medicine, Dr. Dhondge's Clinic, Nashik, IND

**Keywords:** refractory, pedal edema, chikungunya, covid-19, chikungunya fever

## Abstract

An 86-year-old male presented with fever and joint pain for seven days. Clinical features were suggestive of chikungunya fever, but reverse transcription-polymerase chain reaction (RT-PCR) was negative. After ruling out the differentials, the patient was clinically diagnosed with chikungunya fever. Chikungunya IgG antibody was positive two months after the onset of symptoms. He had a history of asymptomatic coronavirus disease (COVID-19) infection two months ago. About 20 days after his first symptom, he developed bipedal edema, refractory to diuretics. All other causes of pedal edema, including heart failure, renal failure, and liver failure, were ruled out. The bipedal edema was managed conservatively with compression bandages. Only a few case reports and studies on limb edema as a symptom post chikungunya fever have been published up to this point. Furthermore, it is difficult to say whether his COVID-19 infection is linked to the edema.

## Introduction

Chikungunya fever is caused by the chikungunya virus, an arbovirus of the Alphavirus genus transmitted to humans by bites of female mosquitoes of the Aedes genus [1]. High fever, malaise, arthralgia, headache, and gastrointestinal complaints are typical clinical manifestations of chikungunya fever [2]. Chikungunya fever has several uncommon presentations, including neurological, vascular, skin, and neonatal variants [3]. Despite their rarity, a limited number of these manifestations can be found in the literature [4,5]. Until now, vascular involvement in chikungunya fever has been mentioned seldom in the medical literature and is often limited to Raynaud's phenomenon that persists beyond the acute phase [6]. Only a few case reports and research on limb edema as a manifestation of chikungunya fever have been published. These individuals had enzyme-linked immunosorbent assay (ELISA)-positive antibodies after acute and subacute infection [7]. In contrast, we had a patient who was COVID-19 positive but did not have pulmonary involvement and was seronegative for chikungunya fever after five weeks of symptom start but was later found to have IgG after two months. We discuss this case in great detail ahead.

## Case presentation

**Figure 1:**
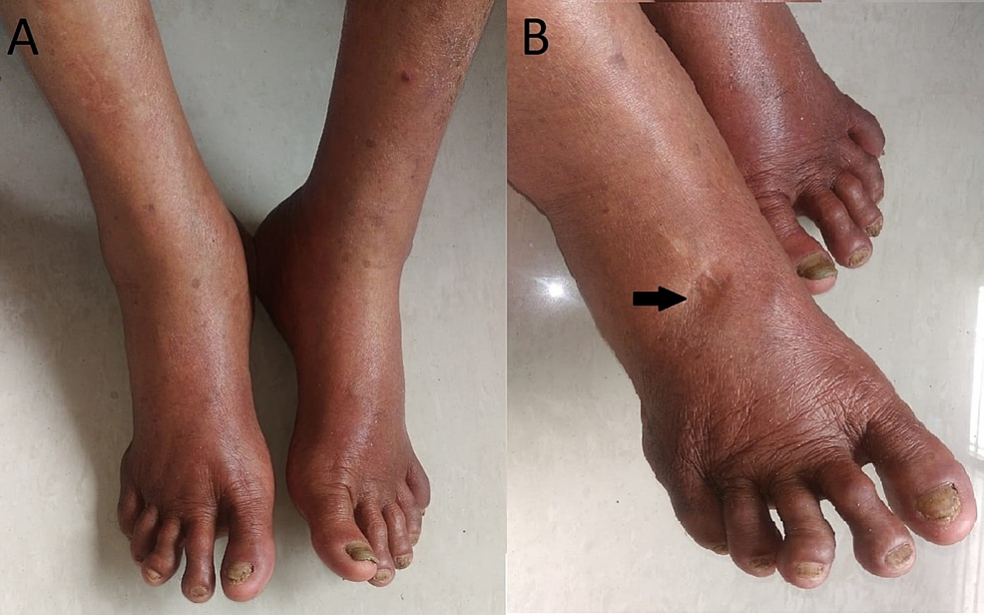
(A) Bilateral pedal edema. (B) Pitting phenomenon.

An 86-year-old male came to the outpatient department (OPD) in June 2021 with a fever (temperature - 102°F) and joint pain complaints for seven days. Fever was insidious in onset, high grade, followed a saddleback pattern, and was associated with chills. The joint pain was insidious in onset and migratory, involving multiple hands, feet, elbow, and knee joints. It was associated with early morning pain and swelling in the hands and feet. One day before visiting the OPD, he developed a pruritic, maculopapular rash that affected the lower limbs, followed by the trunk. He also complained of myalgia, headache, photophobia, vague abdominal pain, nausea, and decreased appetite. There was no history of bleeding, epistaxis, or petechiae. Non-contributory socioeconomic history There is no history of allergies or medication abuse, nor has there been any surgery in the past. There was no previous history of similar illnesses. The patient was hospitalized for five days till the fever was resolved, and post-discharge symptomatic treatment including non-steroidal anti-inflammatory drugs (naproxen 250 mg tablet twice daily) was given for arthralgia and myalgia. Bed rest was advised. The drugs relieved his pain. 20 days after the onset of the joint pain and fever, he developed bilateral pitting edema of the leg (Figures 1A, 1B). The edema was refractory to treatment. Thiazide diuretics (metolazone 5 mg tablet) were administered weekly for 10 doses, but they did not reduce edema in the legs.

**Figure 2:**
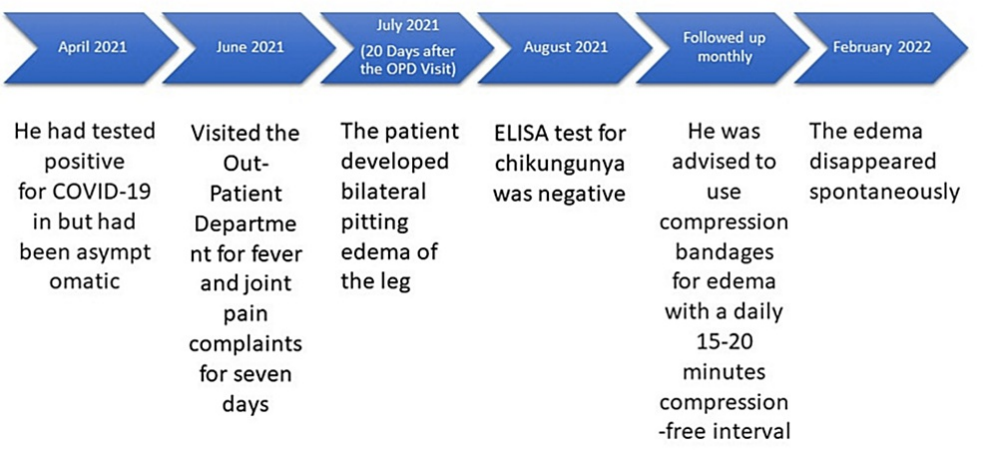
The case timeline

The timeline of the case is depicted in Figure 2. He had tested positive for COVID-19 in April 2021 but was asymptomatic. He resided in a part of India, an area where chikungunya is considered endemic. RT-PCR for the chikungunya virus was negative. ELISA test for chikungunya done at five weeks was negative. However, another done at two months was positive for IgG antibodies. Renal function tests and liver function tests were within normal limits. Echocardiography showed no abnormalities. Doppler ultrasound showed no abnormality in the velocity of the blood. It showed effusion at both knee joints and lymphatic edema extending up to the ankle in both feet. The serum protein electrophoresis showed normal albumin and globulin levels. His biochemical profile is depicted in Table 1. Lymphoscintigraphy was not done.

**Table 1:** Biochemical profile of the patient

Parameter	Value	Reference Value
Leucocytes	7100/µL	4,500 to 11,000/µL
Neutrophils	76%	55-70%
Lymphocytes	17%	20-40%
Eosinophils	3%	1-4%
Monocytes	4%	2-8%
Basophils	0%	0.5-1%
Erythrocytes	5.18 x 10^6^/µL	4.7 to 6.1 x 10^6^/µL
Hemoglobin	15.4 g/dL	13.2 to 16.6 g/dL
Hematocrit	47.1%	38.3 to 48.6 %
Mean corpuscular volume	90.9 fL	80-100 fL
Mean corpuscular hemoglobin	29.7 pg	27.5-33.2 pg
Mean corpuscular hemoglobin concentration	32.7 g/dL	31-37 g/dL
Platelets	129 x 10^3^/µL	150 x 10^3^ – 450 x 10^3^/µL
Serum uric acid	5.6 mg/dL	2.5-7 mg/dL
Serum albumin	4.4 g/dL	3.4 to 5.4 g/dL
Serum alpha 1 globulin	0.11 g/dL	0.1 to 0.3 g/dL
Serum alpha 2 globulin	0.7 g/dL	0.6 to 1 g/dL
Serum beta globulin	0.8 g/dL	0.7 to 1.2 g/dL
Serum gamma globulin	1.3 g/dL	0.7 to 1.6 g/dL
Serum albumin/globulin ratio	1.51	1.1 to 2.5
Serum bilirubin (total)	4 µmol/L	1.71 to 20.5 µmol/L
Serum alkaline phosphatase	47 IU/L	44 to 147 IU/L
Serum aspartate transaminase	29 U/L	8 to 33 U/L
Serum alanine transaminase	31 U/L	7 to 56 U/L
Gamma-glutamyltransferase	38 U/L	5 to 40 U/L
Prothrombin time	14 seconds	11.0 to 12.5 seconds

We kept chikungunya, dengue, malaria, alphavirus infection, rickettsia, group A streptococcus infection, leptospirosis, zika virus infection, West Nile fever, septic arthritis, and rheumatological conditions on our differential. Pitting edema could be caused by polyarthritis, inflammation, congestive cardiac failure, left ventricular failure, pericarditis, inferior vena cava (IVC) obstruction, deep vein thrombosis (DVT), anemia, hypoalbuminemia, beriberi, liver failure, or kidney disease.

Negative serological tests ruled out dengue. Normal findings in echocardiography helped rule out cardiac causes like heart failure. Normal doppler ultrasound ruled out deep vein thrombosis. Normal liver function tests and albumin levels helped rule out liver pathology and hypoalbuminemia. Normal renal function tests and the absence of other features like puffiness of the face and signs of uremia ruled out kidney disease. No signs of infection were present except fever during the onset of symptoms, allowing us to rule out other infectious causes. Normal serum urate levels rule out gout. IVC obstruction was suspected but was ruled out as other relevant examination findings like hypotension, tachycardia, and tachypnea were absent. Moreover, spontaneous flow with adequate phasicity in the external iliac vein and common femoral vein rules out IVC obstruction. His venous doppler study showed effusion at both knee joints and lymphatic edema in both feet extending up to the ankle. We also kept arthropathies our differentials. Seronegative spondyloarthropathies usually manifest before the age of 45 years, which is therefore deemed unlikely in our 86-year-old patient. Other signs like uveitis, inflammatory bowel disease, HLA-B27 negativity, and absence of family history for spondyloarthritis (SpA) or psoriatic arthritis (PsA) made these diagnoses unlikely. Radiological features did not support this diagnosis. Other differentials were ruled out with the help of the biochemical profile.

The patient was then followed up monthly. He was advised to use compression bandages and limb elevation for edema with a daily 15-20 minutes compression-free interval. The edema disappeared spontaneously at the eight-month visit.

## Discussion

Pitting edema of bilateral lower limbs in a patient with chikungunya fever is a rare finding which could be due to many contributing factors [7,8]. There are few resources in the medical literature that have described peripheral edema and explored the causes.

Chikungunya viremia lasts only for approximately one week after the onset of the disease [9]. Ig-M antibodies develop 5-7 days after onset of disease. If RT-PCR is done after six days of onset, there may not be enough viral components in the blood to be detected. The test's sensitivity also plays a role in the validity of the test results. A study showed that the RT-PCR with 856 probe/primer set was more sensitive for the Asian population when compared with the 3566 probe/primer set, which was efficient in detecting more genotypes (East African South Asian - ESCA and Asian) but less sensitive [9]. According to the Centers for Disease Control and Prevention diagnostic testing algorithm, if RT-PCR is negative or presentation is beyond six days of onset of symptoms, the clinical approach requires testing for IgM antibodies in the blood. Many tests are available for the same, including lateral flow rapid tests, IgM antibody capture enzyme-linked immunosorbent assay (MAC-ELISAs), and indirect immunofluorescence assays [9]. Each commercially marketed test has variable sensitivity, and tests with lower sensitivity can cause false-negative results. If both RT-PCR and MAC-ELISA are negative, they are still concluded as inconclusive and need further testing [9].

Lymphedema could have been a possible cause of edema in this patient since his venous doppler study showed effusion at both knee joints and lymphatic edema in both feet extending up to the ankle. The exact mechanism of this lymphedema is not clear. Lymphedema may be caused by the involvement of lymphatics and interstitial space or lymphangitis or increased lymph production, secondary to systemic inflammation [10,11]. Decreased limb movement due to joint pain can decrease lymph circulation [12]. It can be seen early or late in chikungunya patients with swelling, tenosynovitis, or arthritis [13]. Some studies have explored the roles of the immune system, inflammatory mediators like interleukin-6, and even cryoglobulinemia as contributing factors [7,14].

While there is a lot to learn about COVID-19 yet, this case highlights the fact that COVID-19 infection can develop even in persons who have been vaccinated against it, as reported in the literature before. [15] While it is believed that the vaccination can lessen the severity of the infection, if not completely prevent it [16], it is first needed to know all the possible manifestations of the disease to compare the severity of symptoms pre and post-vaccination. This opens the door for additional research to report on and bring these hidden traits to light. Table 2 depicts cases of pedal edema published in the literature linked to chikungunya fever or COVID-19.

**Table 2:** Cases of pedal edema published in the literature linked to chikungunya fever or COVID-19

Case report/Study	Clinical features	Remarks
Pedal edema with/after Chikungunya fever		
Kumar JC et al. [17]	High fever, erythema on the ear, severe polyarthritic joint pains & swelling, non-pitting pedal edema, facial puffiness, and itching for the past four days.	Oral candidiasis in Chikungunya viral fever
Bhat RM et al. [18]	The study found pedal edema in 5.33% of patients with “suspected” chikungunya fever.	Other features included skin rash, aphthae-like ulcers, pigmentary changes, desquamation, exacerbation of the existing dermatoses, urticaria, non-intertriginous necrotic cutaneous ulcers, scrotal dermatitis, pedal edema, and vesiculobullous eruption.
Pedal edema with/after COVID-19		
Sharma and Kar [19]	They studied nine cases that had recovered completely from COVID-19 but developed pedal edema, which could not be attributed to any other organic cause.	Similarity to the present case is that the pedal edema occurred post-recovery.
Pedal edema with/after COVID-19 and Chikungunya		
Our case	Primarily fever and joint pain complaints. Early morning pain and swelling in the hands and feet, pruritic & maculopapular rash, myalgia, headache, photophobia, vague abdominal pain, nausea, and decreased appetite were observed.	It is still unclear if this was lymphedema triggered by the chikungunya virus or peripheral edema caused by COVID-19 or chikungunya.

Given the patient's recent history of asymptomatic COVID-19 infection, the question of it being a contributor to the pedal edema did arise. There is no literature, however, that gives a definitive answer. Acute and transient peripheral edema was reported in some symptomatic cases of COVID-19 and was associated with pain and involvement of overlying skin with the presence of extravasated serum and erythrocytes [20]. However, in our patient, these specific findings were not present and the onset of pedal edema was delayed. It is still unclear if this was lymphedema triggered by the chikungunya virus or peripheral edema caused by COVID-19 or chikungunya.

## Conclusions

There are many known etiologies of bipedal edema in the elderly, including infectious, non-infectious, and post-infectious, but it is important to consider chikungunya fever as another possible etiology. While our patient had a recent history of both COVID-19 and chikungunya infection, it is difficult to comment upon the exact cause of the edema. Conservative treatment was chosen as diuretics did not improve his pedal edema. Sharing the new information about this manifestation among primary care physicians, including infectious disease specialists, can aid in diagnosing and treating these patients accordingly.
